# Segmentation of the Clustered Cells with Optimized Boundary Detection in Negative Phase Contrast Images

**DOI:** 10.1371/journal.pone.0130178

**Published:** 2015-06-12

**Authors:** Yuliang Wang, Zaicheng Zhang, Huimin Wang, Shusheng Bi

**Affiliations:** 1 Robotics Institute, School of Mechanical Engineering and Automation, Beihang University, Beijing 100191, P.R. China; 2 Department of Materials Science and Engineering, The Ohio State University, 2041 College Rd., Columbus, Ohio 43210, United States of America; UGent / VIB, BELGIUM

## Abstract

Cell image segmentation plays a central role in numerous biology studies and clinical applications. As a result, the development of cell image segmentation algorithms with high robustness and accuracy is attracting more and more attention. In this study, an automated cell image segmentation algorithm is developed to get improved cell image segmentation with respect to cell boundary detection and segmentation of the clustered cells for all cells in the field of view in negative phase contrast images. A new method which combines the thresholding method and edge based active contour method was proposed to optimize cell boundary detection. In order to segment clustered cells, the geographic peaks of cell light intensity were utilized to detect numbers and locations of the clustered cells. In this paper, the working principles of the algorithms are described. The influence of parameters in cell boundary detection and the selection of the threshold value on the final segmentation results are investigated. At last, the proposed algorithm is applied to the negative phase contrast images from different experiments. The performance of the proposed method is evaluated. Results show that the proposed method can achieve optimized cell boundary detection and highly accurate segmentation for clustered cells.

## Introduction

Cell image segmentation is a process which differentiates cell regions from the background in images containing one or more cells. It plays an important role in both fundamental biology research [1–3] and clinical applications [4] regarding cell morphology analysis and cell behavior characterization. Cell image segmentation is at the center of many applications, such as drug development [5], pap smear test [6], cell classification and cell phase detection [7]. Cell image segmentation is also a crucial step for cell tracking, which is widely applied in characterizations of cell behaviors, including directed cell migration [8–10], wound healing [11], and tumor cell metastasis and invasion [12, 13].

Cell image segmentation can be performed either manually [14, 15] or automatically [16–18] for the acquired images. Since cells are live objects and cellular processes are normally stochastic [19], the analyses mostly relay on the massive measurement of hundreds or even thousands cells in a single experiment. As a result, high throughput image screening obtained with time-lapse microscope imaging is widely applied in cell biology measurement [20]. The manual processing of the high-throughput image sequences is extremely time-consuming. Therefore, automated cell image segmentation is generally applied.

Technically speaking, automated cell image segmentation includes two aspects, cell localization and cell boundary detection. Cell localization is a process of determining cell location in cell images. It is essential for cell migration related studies. Cell boundary detection is a process of extracting contours which are as close as possible to cell actual boundaries. The accuracy of cell boundary detection is important for cell morphology related studies. Multiple algorithms have been applied to achieve automated cell image segmentation in acquired cell images, including thresholding methods [17, 20, 21], active contour methods [16, 18], and level set methods [22–25]. Each of them can realize cell image segmentation to some extend with combination of different cell imaging techniques or image pre-processing algorithms, like Gaussian kernel convolution [20, 26] and Bhattacharyya transform [27]. However, improper cell image segmentation may cause oversegmentation (a cell is falsely fragmented as two or more cells) or undersegmentation (two or more cells are detected as one) in cell image segmentation.

The performance and methods applied in automated cell image segmentation are strongly related to cell imaging techniques. Many cell imaging techniques are applied to get cell images with improved image contrast [14, 18, 23, 28–31]. Of all the methods, fluorescence imaging and phase contrast imaging (positive phase contrast, more specifically) are two widely applied techniques. Fluorescence imaging provides good image contrast. However, it normally suffers from photobleaching, which limits its applications in long term cell monitoring. Moreover, in fluorescence imaging, cells need to be either genetically engineered to generate fluorescent proteins or fluorescently labeled to enhance cell boundary information, which modifies cell physiological makeup and may cause unknown change of cellular dynamics. Positive phase contrast images provide relatively high image contrast without any biological modification to cells, which makes it a good alternative for cell image segmentation [14, 18, 30, 32–34]. In positive phase contrast images, cell bodies normally show lower light intensity than the background. However, cells with increased cell height (like mitotic cells) show reversed image contrast such that their bodies have higher light intensity than background. As a result, one needs to segment cells with low and high light intensity separately in a two-step approach [30].

Currently people are facing several challenges in cell image segmentation. First, the cell boundary detection for massive cells in the field of view needs to be optimized. Most of cell image segmentation algorithms focus on cell localization. Recently, the optimization of cell boundary detection is getting more and more attentions [32–36]. In the segmentation with fluorescence images, only nuclei are generally stained and segmented, leaving cytoplasm undetectable [37]. To get the whole cell image segmentation (nucleus + cytoplasm), it requires the combination of a separate staining of actin filaments in another channel [26, 38] or complex bright field image stacking [29]. Some other whole cell image segmentation methods with fluorescence images strongly relay on the presence of the bright conjunction lines between two contacting cells, which normally exist in tissue [36] or confluent cell monolayers [38]. For the positive phase contrast images, the region based active contour method can automatically segment all cells in the field of view which is applicable for high-throughput image processing [16, 30]. Edge based active contour method utilizes the local light intensity information and can achieve a good cell boundary detection. However, this method normally needs contour initialization which is tedious when large number of cells need to be segmented [18]. Ersoy et al. utilized a ridge detection method to detect halos around cell bodies in positive phase contrast images and obtained a good boundary estimation [35]. It requires the detection of inner and outer edges of halos and may not work efficiently when cell confluence is high. Plus, the halos are not consistent from frame to frame in the positive phase contrast images [21].

Recently, several two-step approaches are proposed to achieve optimized boundary detection [32, 33]. In a method proposed by Seroussi et al. (2012)[33], they first applied the gradient vector flow (GVF) based active contour method to get approximate cell boundaries. After that, a directional GVF field is constructed by considering only image light intensity gradient pointing outwards with respect to the approximate cell boundaries obtained in the first step. In a two-step algorithm proposed by Chalfoun et al.[32], they first detect seed points either through histogram quantization of light intensity or biological modeling of nucleoli within cell nucleus areas. After that, the individual pixels are assigned to the seed points which have shortest geodesic distance to the unassigned pixels.

Second, algorithms with efficient segmentation of clustered cells need to be developed. Many cells have intention to contact each other and form crump areas. Current cell image segmentation algorithms have difficulty in accurately segmenting the contacting cells. The segmentation of the contacting cells needs to solve two problems. One is the detection of cell numbers and cell locations within clump areas containing multiple cells. The other is the segmentation of the cytoplasm areas for contacting cells. Numerous approaches have been proposed in the segmentation of clustered cells. They can be categorized as shape based or marker based approaches. The shape based approaches utilize the characteristic geographical structures, like concave vertex [37] or symmetry properties of boundaries [39, 40], to segment the clustered cells. The marker based approaches first detect markers within the clump areas. Individual cells are localized with the detected markers. The generation of the markers mostly relays on the distance transform of the preliminarily identified cell areas, where the local minima are taken as markers. This is widely applied in the so called watershed method [7, 28, 41]. The watershed method often causes over-segmentation and may need complex merging algorithms [32, 42, 43] or by combination of Euclidean distance transform and light intensity information in the areas containing clustered cells [34]. Additionally, even with the successful cell localization in a clump area, current algorithms still could not optimize the detection of the actual boundaries between any two contacting cells. The contacting cells may be simply divided along the ridge in the distance transform.

In this study, our goal is to provide a systematic approach for cell image segmentation in terms of cell imaging, cell boundary detection, and separation of the clustered cells. As mentioned earlier, in the positive phase contrast images, cells with increased height may show reversed image contrast. To solve this issue, the negative phase contrast images were used. In the negative phase contrast images used in this study, cell bodies consistently show high light intensity and contrast reversion does not happen for cells with increased height. The cell boundary detection was achieved through the combination of global and local based approaches. In the global approach, the thresholding method was applied to get a preliminary segmentation result for all cells in the field of view. Then, the contours extracted in the preliminary segmentation results were taken as the initial guesses to implement the edge based active contour method for individual cells. By doing this, the automated cell localization and optimization of cell boundary detection were achieved. Regarding segmentation of the clustered cells, we utilize cell light intensity information rather than shape of the preliminarily detected areas to optimize cell image segmentation. The geographic peaks of light intensity within the detected areas were used to determine the numbers and locations of multiple cells and their corresponding boundaries were determined using a modified edge based active contour method.

The rest of this paper is organized as follows. In section 2, we introduce the experimental details, including preparation of cell samples, experimental setup and cell imaging techniques. In section 3, the algorithms for cell boundary detection and segmentation of the clustered cells are presented in detail. In section 4, the influence of different parameters on cell image segmentation result is first discussed. Then the proposed cell image segmentation method was applied to images acquired from four experiments.

## Experimental

### Cell culture

In this study, an established non-tumorigenic breast epithelial cells MCF 10A [44, 45] were obtained from the American Type Culture Collection (Manassas, VA, USA). MCF 10A cells were maintained in 47.5% Dulbecco’s modified Eagle’s medium (DMEM) and 47.5% F-12 medium supplemented with 5% horse serum, EGF (20 ng/ml), bovine insulin (1*μ*g/ml), hydrocortisone (0.5 *μ*g/ ml), Cholora toxin (0.1*μ*g/ml), NaHCO_3_ (0.2 mM), and 1% penicillin/streptomycin.

During imaging, cells were placed on a stage-top incubator (Model: WSKM-F1, Tokai Hit, Japan) with controlled humidity and medium temperature (37°C). The pH value of culture media was maintained by connecting the stage-top incubator with the pre-mixed air with 5% CO_2_ supplied through a CO_2_ controller (Model No.: DGTCO2BX, OKOLab, Italy). With the above setup, our tests show that cells can be incubated for more than three days.

### Cell imaging and image acquisition

Before imaging, cells were seeded in six-well plates for 24 hours. After that, samples were rinsed with fresh medium to remove debris that may interfere imaging. Then the plates were transferred to the stage-top incubator for monitoring. The negative phase contrast imaging was applied in this study by using a phase contrast microscope (Model: IX51, Olympus). A 10X negative phase contrast lens (Model: PLN10XPH/NH, Olympus) was used to get negative phase contrast images. A CCD camera (Model: C4742-95, Hamamatsu, Japan) was used for image acquisition. The image acquisition was implemented through the software Wasabi (Version 1.5, Hamamatsu, Japan). The pixel size for all grabbed images in this study is 1344 ×1024, which corresponding to an actual field of view of 900 *μ*m× 686 *μ*m.

**Fig 1:**
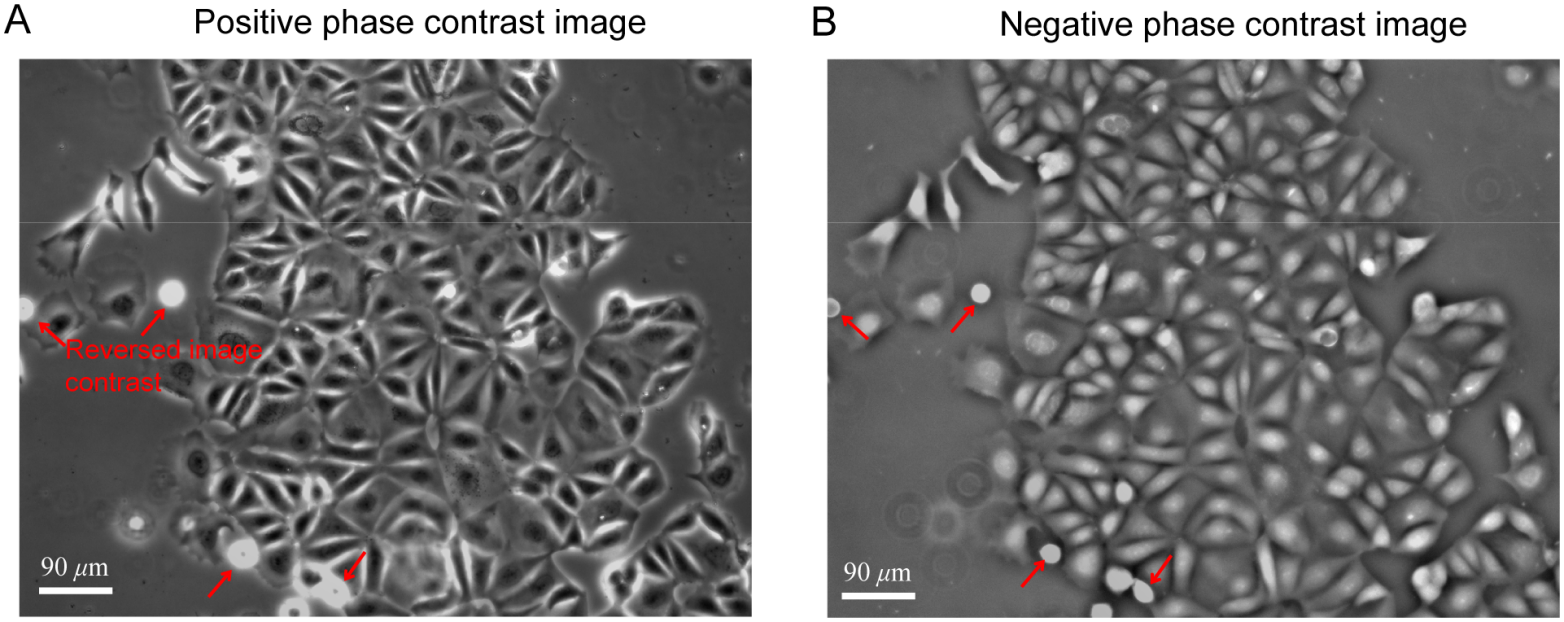
Comparison of positive (A) and negative (B) phase contrast images of the MCF 10A cells. In the positive phase contrast image, cells with larger height show reversed image contrast, while in the negative phase contrast image, all cells have consistent image contrast.

A comparison of positive and negative phase contrast images is shown in Fig 1. In the positive phase contrast images shown in Fig 1A, the cell body normally has lower light intensity than background. Mitotic cells as well as cells with higher cell height show the reversed image contrast. Several mitotic cells (marked by red arrows) show much higher light intensity than the background. In the negative phase contrast images, all cells including mitotic cells consistently show positive image contrast, as indicated in Fig 1B. This eliminates the image contrast reversion caused by increased cell height. Four different sets of experiments were conducted and images were taken after incubation in the stage top incubator for more than 20 hours to make sure cells were fully settled. Images with moderate cell density of 120–150 cells per field of view (900 *μ*m× 686 *μ*m) and coverage areas of 12%- 20% were used for cell image segmentation.

## Methods in Cell Image Segmentation

In this section, methods for boundary detection and segmentation of the clustered cells will be presented one by one.

### Cell boundary detection

**Fig 2:**
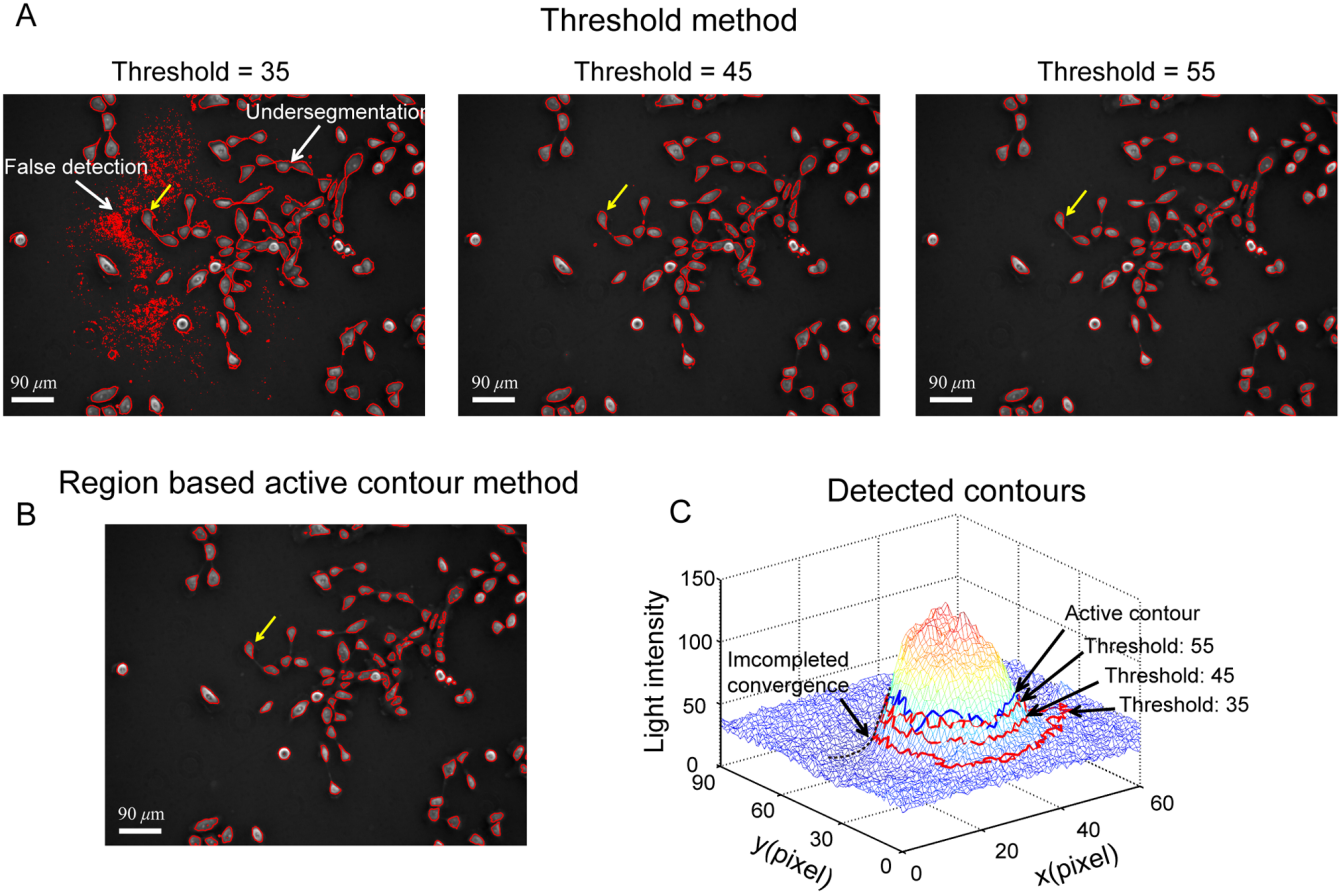
Cell image segmentation result obtained with different methods. (A) In the thresholding method, the segmentation result is sensitive to the selection of the threshold value. The detected contours shrink with increasing threshold value. (B) Cell image segmentation with the region based active contour method. (C) Comparison of the contours obtained with the region based active contour method and the thresholding method with different threshold values. It is apparent that both methods underestimate cell actual boundary.

Thresholding and region based active contour methods are two popular methods in automated cell image segmentation. Fig 2A shows the segmentation results obtained using the thresholding method with different threshold values. For a low threshold value (35, in this case), one can see that lots of substrate areas were falsely detected as cell areas and undersegmentation occurs for some cells, as pointed by the white arrows in the first image of Fig 2A. With the increasing threshold values (45 and 55, in this case), false detection of substrate areas disappears and a better segmentation result is achieved, as shown in the second and third image in Fig 2A. In addition to the thresholding method, the region based active contour method was also applied to the cell image. The image segmentation result with the region based active contour method is shown in Fig 2B.

Fig 2C shows the detected contours obtained with the thresholding method and region based active contour method for a cell pointed by yellow arrows in Fig 2A and 2B. It is apparent that the detected contours are sensitive to the selection of the threshold values in the thresholding method. The areas enclosed by the detected contours decrease with increasing threshold values. Even for the contour obtained with the low threshold value, it still could not converge to cell boundary, as indicated by an arrow in Fig 2C. Similarly, since it is still a global based method, the contour obtained with the region based active contour method could not reach the actual cell boundary.

To get the optimized cell boundary detection, a new approach which is referred to as ***contour expansion method*** is introduced in this paper. The method utilizes the information of the light intensity distribution over cell surface. In a negative phase contrast microscopy, the phase shift Δ*ϕ* caused by the presence of phase objects can be given as:

$$\Delta \phi =2\pi (n_{2}-n_{1})h/\lambda ,$$ (1)

where *n*_1_ and *n*_2_ are refractive indexes of the surrounding media and phase objects, respectively, *h* is the thickness of the phase objects, *λ* is the wavelength of illumination light. The relationship between light intensity *I* and the object-induced phase shift Δ*ϕ* can be given as [46]

$$I={\left|\text{exp}[j(\pi /2)]+j\Delta \phi \right|}^{2}={\left|j(1+\Delta \phi )\right|}^{2}\approx 1+2\Delta \phi .$$ (2)

**Fig 3:**
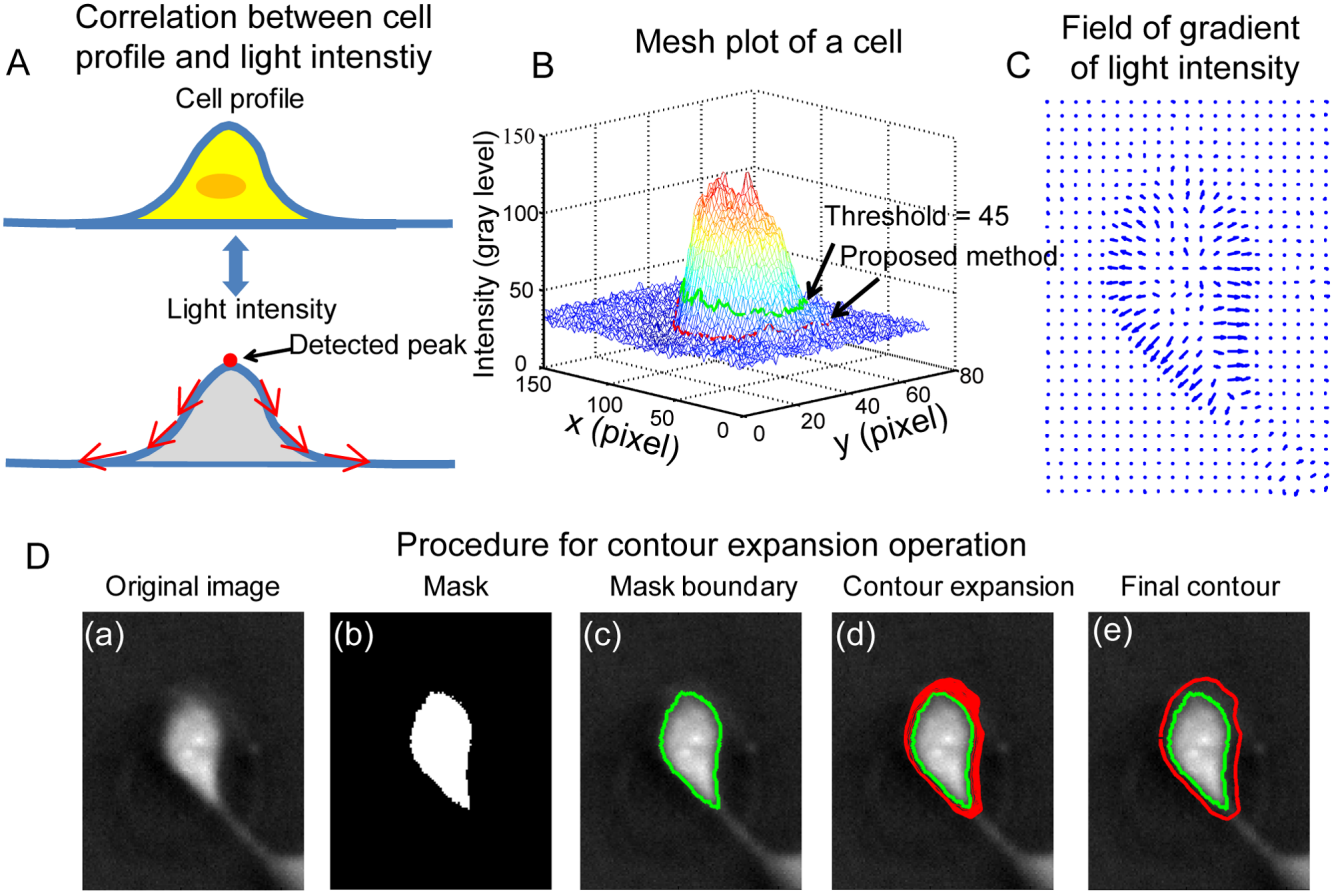
Contour expansion method for cell boundary detection. (A) It is assumed that cells have illustrated height profile with one peak located above cell nucleus. In negative phase contrast images, the light intensity of cells is proportional to cell height. Therefore, the light intensity distribution over cell surface is similar to height profile of cells with one peak located above each cell body. (B) Mesh plot of the light inteisity for a selected cell marked with yellow arrows in Fig 2A and 2B. The light intensity decreases towards the cell boundary, which is consistent with the illustration shown in (A). (C) Quiver plot of the gradient of light intensity for the selected cell. Over cell surface, the gradient of light intensity pointing outwards. (D) The procedure of the contour expansion method for cell boundary detection: (a) The raw negative phase contrast image of the selected cell. (b) The thresholding method was used to get a preliminary mask for the selected cell. (c) The boundary of the mask was extracted and taken as the initial contour. (d) With contour expansion method, the initial contour is driven by the field of gradient of light intensity to gradually converge to the cell boundary. (e) The contour is finally converged at the boundary of the cell, where the contour achieves the minimum energy. The comparison of the contour detected with the thresholding method and the proposed method is shown in (B). It is clear that the proposed method provides an improved estimation of the cell boundary.

Therefore, the light intensity is approximately linearly related to the phase shift Δ*ϕ*. From Eq 1, one can see that Δ*ϕ* induced by a phase object is proportional to its thickness. Therefore, one can conclude that the light intensity should also be linearly related to the thickness of the phase objects. Normally, cells have a height profile as shown in the top graph of Fig 3A. The apex of the profile locates above cell nucleus. The height gradually decreases towards cell boundaries. Based on the Eqs 1 and 2, one can expect that the light intensity of the cell in negative phase contrast images should have the similar convex profile over cell surface with the height profile, as illustrated in the bottom graph of Fig 3A.

Fig 3B shows a mesh plot of the light intensity for a negative phase contrast cell image. One can see that light intensity has a higher value in the central area of the cell and then gradually decreases toward cell boundary area, which is consistent with the graph shown in Fig 3A. By taking the differentiation of the light intensity along both x and y directions, the field of gradient of light intensity is obtained, as shown in Fig 3C. The field of gradient can be used to define the outline of cell boundary in 2D culture.

Here the traditional edge based active contour method [47] is adapted to detect cell boundary. The original edge based active contour method requires initial contours which are close to the actual boundaries to get accurate detection. In some studies of cell image segmentation, this was done by manually drawing contours outside cell actual boundaries [18]. In this study, the thresholding method and the edge based active contour method are combined to carry out automated cell image segmentation for all cells in the field of view. Two methods are used to determine the threshold value in the threshold method. One is the mean light intensity based method. The other is the Otsu’s thresholding based method. In the first method, the mean light intensity of the whole image is first calculated. After that, an offset value is added with the obtained mean light intensity and the sum is taken as the threshold. In the second method, a preliminary threshold value is first calculated using the Otsu’s method. Then an offset value is empirically selected to add up with the preliminary threshold. The obtained one is taken as the threshold value.

In the implementation of cell boundary detection, instead of manually drawing the initial contours for cells, we take the contours detected by the thresholding method as the initial guesses for the implementation of the edge based active contour method. The initial contours mostly located within the actual cell boundaries and will expand outwards towards cell actual boundaries. In this study, the method is referred to as the ***contour expansion method***. Fig 3D demonstrates the procedure of the contour expansion method. Fig 3D.a is the negative phase contrast image of a MCF 10A cell. A mask was obtained after applying the thresholding method, as shown in Fig 3D.b. The boundary of the mask (the green contour in Fig 3D.c) is extracted to serve as the initial contour for the contour expansion operation using the edge base active contour method. In the edge based active contour model, a contour in an image is defined as a parametric contour ***v***(*s*) = (*x*(*s*), *y*(*s*)) and has an energy function given as [47]:

$$E=\int_{0}^{1}\left(\frac{1}{2}\alpha v_{s}^{2}+\frac{1}{2}\beta v_{ss}^{2}+E_{ext}\right)ds$$ (3)

where ***v***_*s*_ and ***v***_*ss*_ are first and second order partial derivatives, and *α* and *β* are scalar coefficients. The first two terms in the right side of the Eq 3 is the internal energy of the contour, while the *E*_ext_ represents the external energy of the contour. Here, the light intensity *I* along the contour is taken as the external force. The internal energy depends only on the curve geometry and enforces the continuity and certain smoothness of the curve. The minimization of the total energy *E* satisfies the associated Eular-Lagrange function, given as [47]:

$$\alpha v_{ss}^{}(s,t)-\beta v_{ssss}^{}(s,t)-\nabla E_{ext}=0$$ (4)

where ***v***_*ssss*_ is the fourth order partial derivatives of ***v***(*s*). By iteratively solving the equation, the contour will be deformed and converged towards the cell boundary, where the total energy of the contour is minimized. For the detailed process of numerical solution of the Eq (4), readers can refer to the Supporting Information (S1 File).

Driven by the field of gradient of light intensity, the initial contour expands outward, as indicated by the red contours shown in Fig 3D.d. The contour will stop at cell boundary where it achieves the minimum energy, as shown in Fig 3D.e. A comparison of the contours obtained by the thresholding method and the proposed method is shown in Fig 3B. One can see that the contour obtained with the contour expansion method (red contour) has an improved approximation of the cell boundary than that obtained with the thresholding method (green contour).

**Fig 4:**
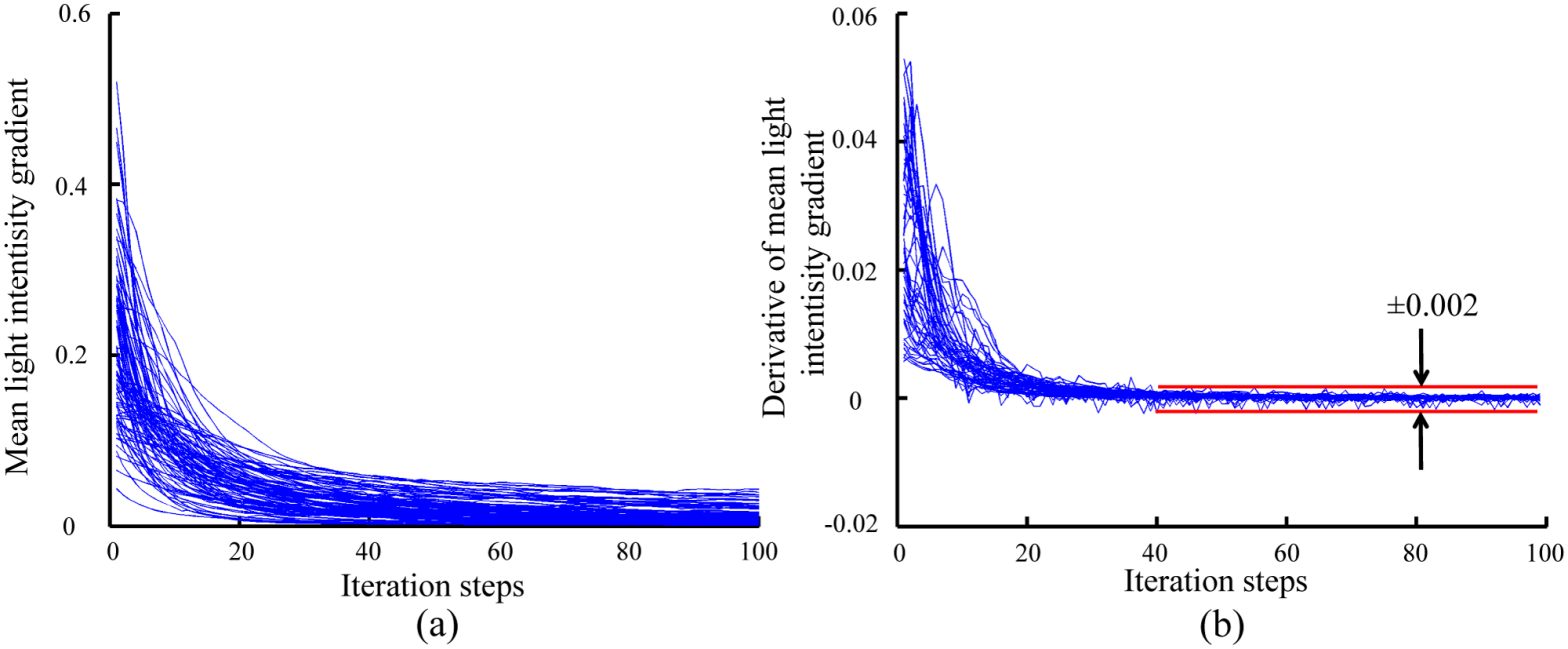
**(A) Variation of the mean light intensity and (B) its time derivative along detected contours during contour expansion.** The mean light intensity gradient decreases with time and mostly reaches its steady state value after about 40 times iteration. The time derivative of the mean light intensity was used as the termination condition for the iteration.

The iterative solution to Eq (4) will lead to the convergence of the initial contours to cell actual boundaries in contour expansion operation. Essentially, the gradient of light intensity is the force which deforms contours. The contour expansion method is applied to cells in the negative phase contrast images shown in Fig 2. The change of the mean value of the light intensity gradient along contours with iteration steps is shown in Fig 4A. One can see that initially the mean light intensity rapidly decreases with increasing iteration steps. After about 40 iteration steps, the mean value becomes steady. In this study, when the difference of mean light intensity gradient between two consecutive iteration steps is lower than 0.002 for over 10 consecutive steps, the iteration will stop and the obtained contours will be taken as the converged contours. Fig 4B shows difference of the mean light intensity gradient value as a function of iteration steps. After about 40 iteration steps, the difference of the mean light intensity gradient decreases to ±0.002.

### Segmentation of the clustered cells

As mentioned earlier, one major challenge in cell image segmentation is the segmentation of clustered cells. Practically, a mask area initialized by the thresholding method (or the region based active contour method) may contain more than one cells. These cells are called clustered cells. Cell image segmentation programs need to detect the number and locations of the clustered cells. The watershed method is the most popular one in the segmentation of the clustered cells.

**Fig 5:**
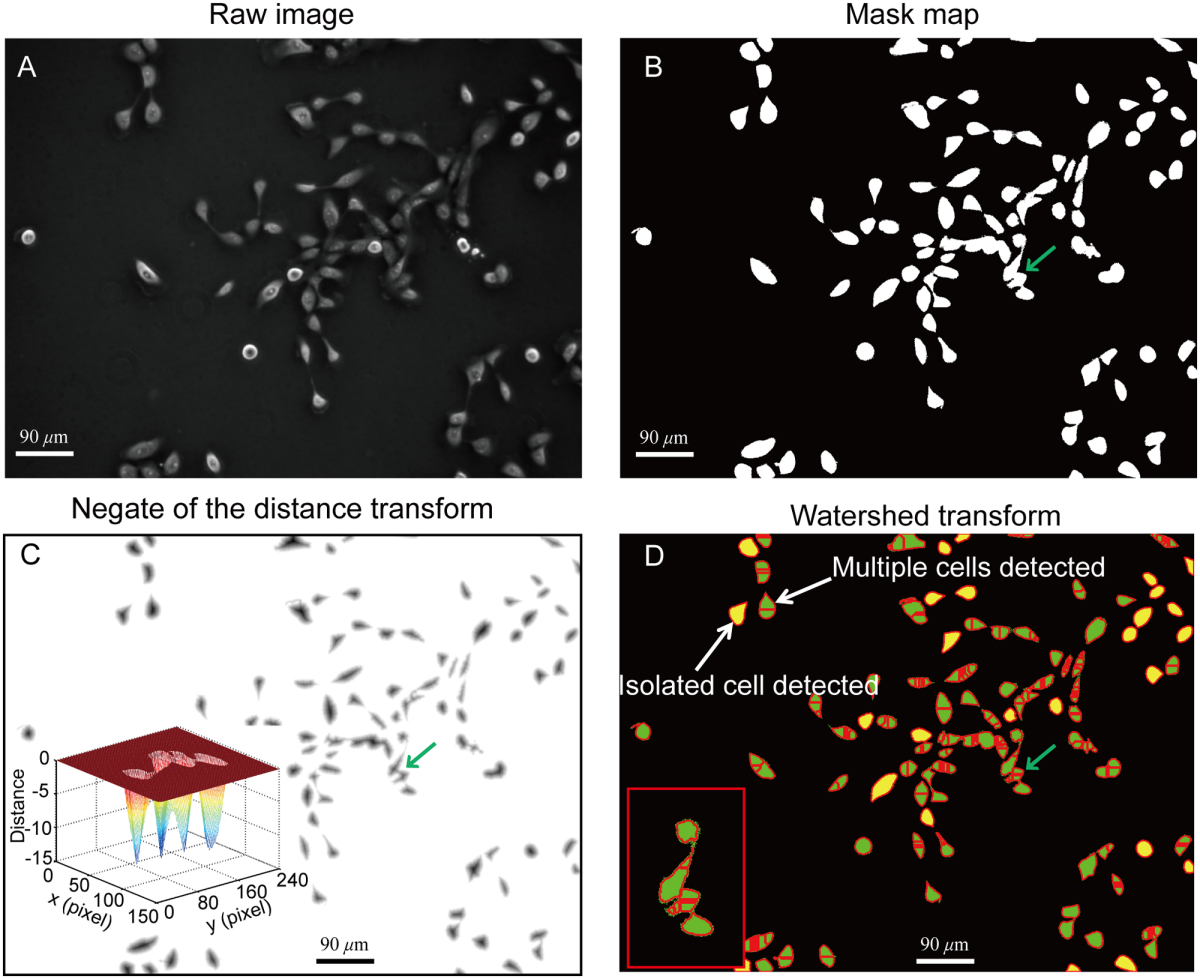
Segmentation of the clustered cells using the watershed method. (A) Raw negative phase contrast image. (B) Preliminarily detected mask map with the thresholding method (threshold = 45). (C) Negate of the distance transform. The inset shows the mesh plot of an area marked by a green arrow. (D) Watershed transform for the detection and segmentation of the clustered cells. The yellow masks are detected isolated cells and the green masks are detected clustered cells. The inset is the enlarged area marked by a green arrow, where four cells are aggregated. The watershed method detected eight cells in the area.

Fig 5 shows the general procedure for the segmentation of the clustered cells with the watershed method. Fig 5A is the raw negative phase contrast image of MCF 10A cells. The thresholding method was applied to the image and a mask map was obtained, as shown in Fig 5B. After that, the Euclidian distance transform was implemented (S1 Fig). To implement the watershed method, the negate of the distance transform was generated, as shown in Fig 5C. The inset shows the mesh plot of a selected area marked by a green arrow in the figure. The watershed method was then applied to the map, as shown in Fig 5D. In the figure, the yellow areas are detected areas with only one cell, while the green areas were detected areas with multiple cells. By comparing Fig 5A and 5C, one can see that more than half of the isolated cells were falsely detected as clustered cells. For the clustered cells, the number and location of cells are mostly falsely detected.

The watershed method strongly relies on the Euclidian distance transform. The generation of Euclidian distance transform is solely determined by the shape of the mask area. The information of cell number and locations may not be well reflected through the shape of the mask areas of the clustered cells. Additionally, even if the number of cells in a mask area was correctly determined with the Euclidian distance transform, the actual boundaries between two contacting cells still could not be accurately detected using the watershed method.

**Fig 6:**
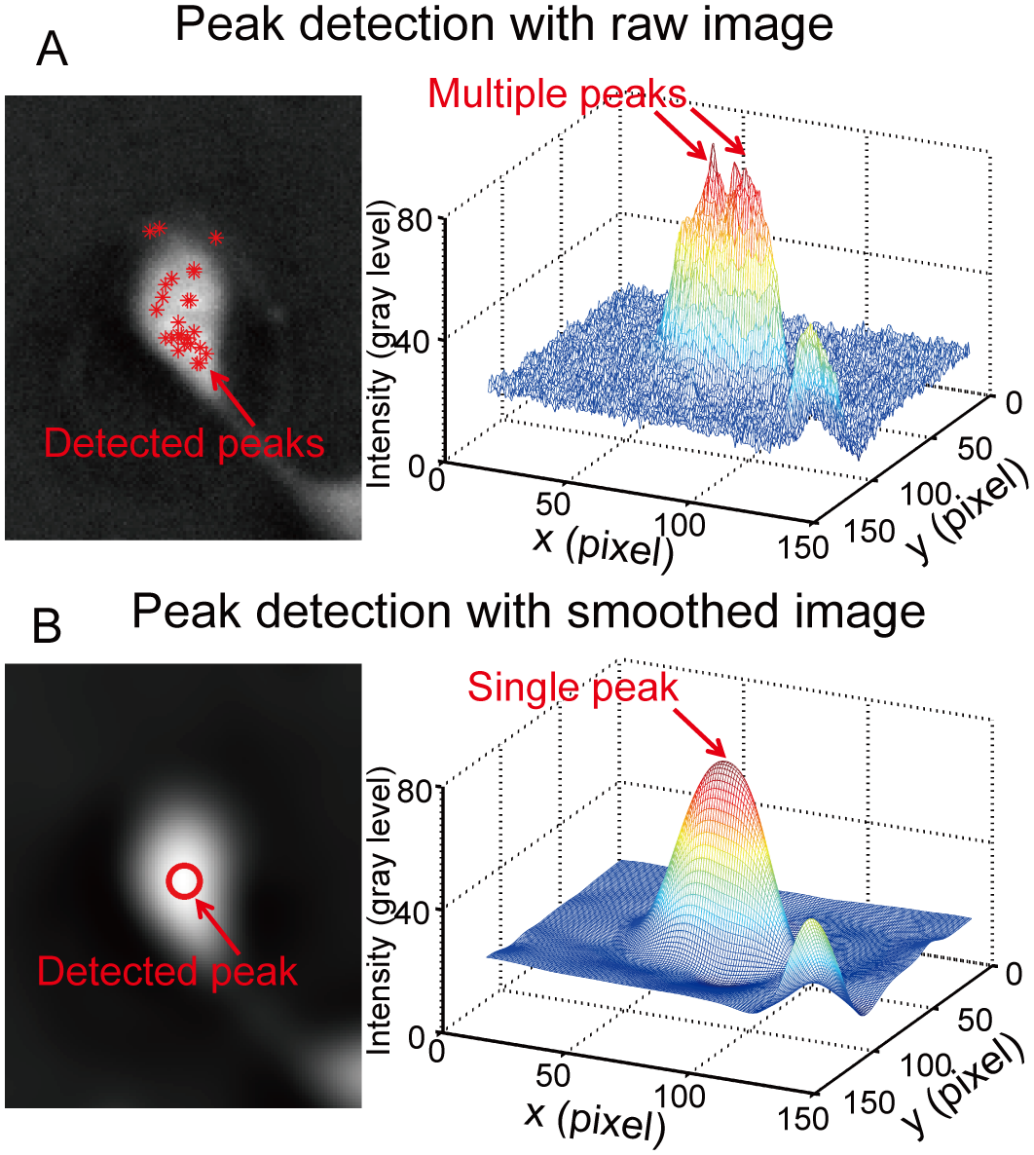
Peak detection for cell localization. (A) Multiple peaks could be detected in a single cell with the raw image due to the existence of bright spots in the image. (B) The image smoothness is implemented prior to the peak detection. With the smoothed image, only one peak is detected.

In this study, instead of using the “shape” information of the preliminarily detected mask areas, we utilize the light intensity to locate cells and detect the boundaries of the clustered cells. As mentioned earlier, the light intensity achieves its higher value around nucleus area and gradually decreases towards cell boundaries in negative phase contrast images. Here we take advantage of the convex shape of light intensity to help to detect cell locations. The “number” and “location(s)” of cell(s) were determined by detecting the number of light intensity peaks in a given mask area. The intensity peaks was located by detecting the regional maximum [48] of light intensity in the negative phase contrast images. With this method, multiple peaks may be detected within a single cell, which is referred to as pseudo-peaks in this study. These pseudo-peaks are mostly caused by the existence of cell organelles. Some organelles may have higher light refractive index than cell cytosol. As a result, they present higher light intensity in the negative phase contrast images. During peak detection, they will be falsely detected as peaks. To get rid of these pseudo-peaks, the image smoothness was implemented to the original phase contrast images [49]. Fig 6A shows the original phase contrast image (left) and its mesh plot (right). One can see that, multiple peaks were detected during peak detection. After image smoothness, the cell image with a single peak was obtained, as shown in Fig 6B.

**Fig 7:**
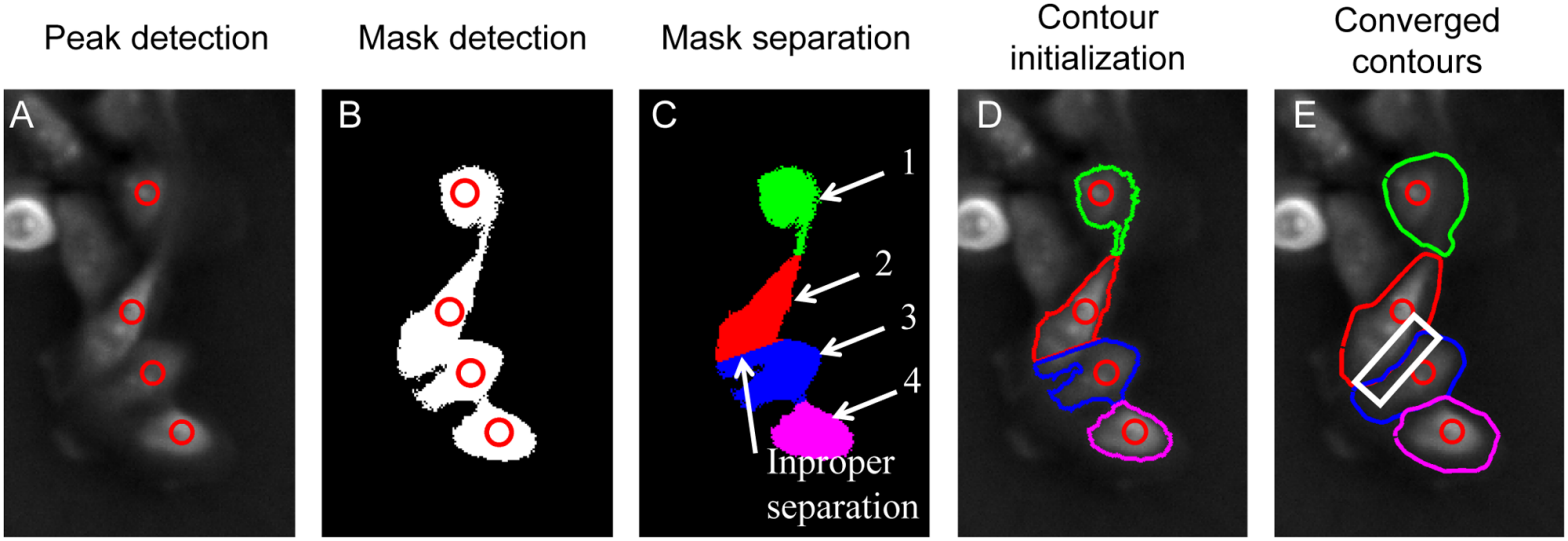
Segmentation of the clustered cells through peak detection. (A) Raw phase contrast image with detected peaks for the clustered cells. (B) Mask area preliminarily detected with the thresholding method. (C) Segmentation of the mask area based on the distance between the pixels and the detected peaks inside the mask area. Each pixel is associated with the peak which has the shortest distance with it. In the figure, the subareas for individual cells are plotted as different colours. (D) Boundaries of the subareas were extracted as the initial contours for contour expansion operation. (E) After contour expansion, the final contours for each cell were obtained with improved estimation of cell boundaries.

In this study, the peak detection method is applied to segment clustered cells. The procedure is demonstrated in Fig 7. For the four contacting cells in Fig 7A, four peaks were first detected with the method mentioned above. After the thresholding method was applied, a mask area containing the clustered cells was obtained. With the detected peaks, the number of cells and their approximate locations in the mask area can be determined, as shown in Fig 7B. To apply the contour expansion operation to the clustered cells, the initial contours must be provided for each cell within the mask area. To do this, the mask area is first divided into several subareas based on the shortest distance between pixels and the detected peaks, as shown in Fig 7C. Each subarea is associated with one clustered cell in the area. Then, the boundaries of these subareas are directly extracted and taken as the initial contours of individual clustered cells for contour expansion operation, as shown in Fig 7D. With these initial contours, the contour expansion method was applied to each cell and the cell boundaries detection is implemented, as shown in Fig 7E.

Note that improper division may occur during contour initialization for the clustered cells. However, most of them can be automatically corrected during contour expansion operation. As shown in Fig 7C and 7D, part of area in cell 2 is falsely assigned to cell 3. During the contour expansion operation, this was automatically corrected, as shown in the selected area in Fig 7E.

In this study, all the algorithms developed and discussed above, including contour expansion method and segmentation of the clustered cells, were implemented using functions developed in Matlab (Version 2012a, The Mathworks, Inc., USA). A developed Matlab tool box as well as raw phase contrast images used in this paper is included in the Supporting Information (S1 Zip File). A README.txt file is included in the tool box, which specifies the usage of each function. All functions in the tool box have been tested in Matlab 2012a. To reproduce the reported results, one just needs to run the functions in the tool box to the included raw images. Note that, a Matlab license may be required to run Matlab functions.

## Results and Discussion

In this section, the influence of the parameters in the contour expansion operation on the final converged contours will be discussed. The proposed method is then applied to cell images from four different experiments. The performance of the cell image segmentation algorithm is evaluated.

**Fig 8:**
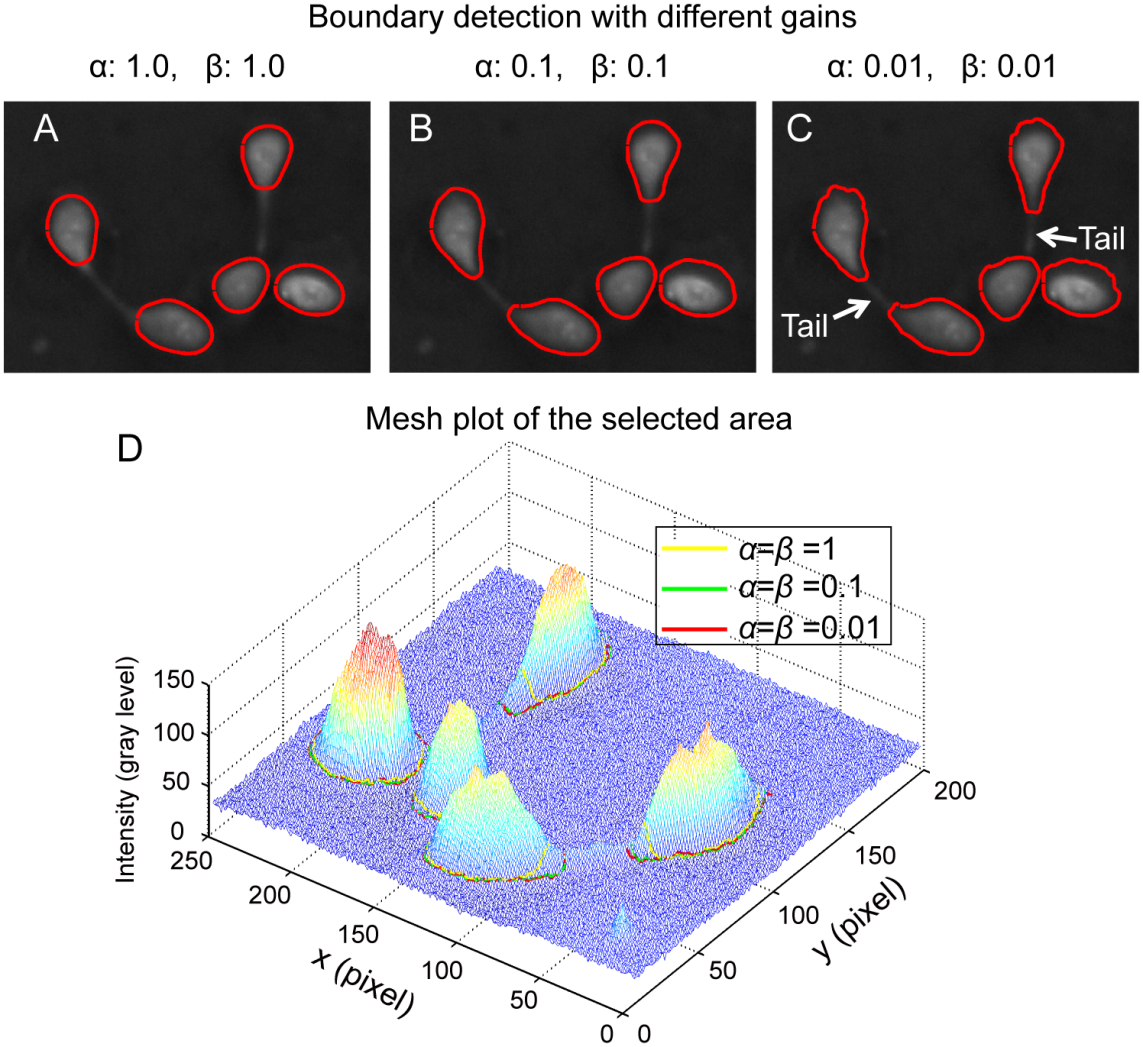
Contour expansion operation with different gains. (A) When the gains *α* and *β* are large, the internal energy dominates the evolution of contours and the contours tends to shrink to minimize the total energy. (B) With decreased gains, the final detected boundaries expanded and were closer to the cell boundaries. (C) When *α* and *β* further decreases to 0.01, optimized estimations of cell boundaries with more details are obtained. (D) Mesh plot of the selected area showing cells and converged contours with different combination of*α* and *β*. The combination *α* = *β* = 1 gives poor estimation of cell boundaries compared with the other two combinations.

Eq 3 indicates that the evolution of an initialized contour is a minimization problem of the total energy. On one hand, the contour tends to shrink to minimize its internal energy. On the other hand, the contour will be pushed towards cell boundaries to minimize its external energy. Practically, the competition of the shrinkage and the expansion is regulated through the parameters *α* and *β* in Eq 3. If higher values were assigned to *α* and *β*, the internal energy will dominate the contour convergence and the contours will not converge into cell boundaries. A comparison of different combinations of *α* and *β* are shown in Fig 8. One can see that, the final contours expand with decreasing *α* and *β*. When *α* and *β* are large, the internal energy of the contour dominates the evolution of contours. The final contour could not converge to cell boundary, as shown in Fig 8A. When *α* and *β* decreases to 0.1, the final contours expand and get more close to cell boundary, as shown in Fig 8B. Our strategy is to get optimized cell boundary detection while maintaining the integrity and avoiding any over estimation of cell boundaries. In this study, *α* and *β* are empirically tuned by checking the performance of the contour detection, either in 2D or 3D meshing plot. By checking the final detected contours in the mesh plot, the combination of α = 0.01 and *β* = 0.01 meets our requirement and is chosen for the boundary detection. The result is shown in Fig 8C. The comparison of the detected cell boundaries with different combinations of *α* and *β* is shown in a mesh plot of Fig 8D. From the figure, one can see that the contours obtained with the combination of *α* = 1 and *β* = 1 encloses much less areas than the other two combinations. From Fig 8C, one can see that the proposed method is effective in capturing cell bodies and gives optimal cell boundary detection. However, due to the constraint of internal energy, the contours could not converge to sharp protrusion structures like tails of cells, as pointed by two arrows in Fig 8C.

**Fig 9:**
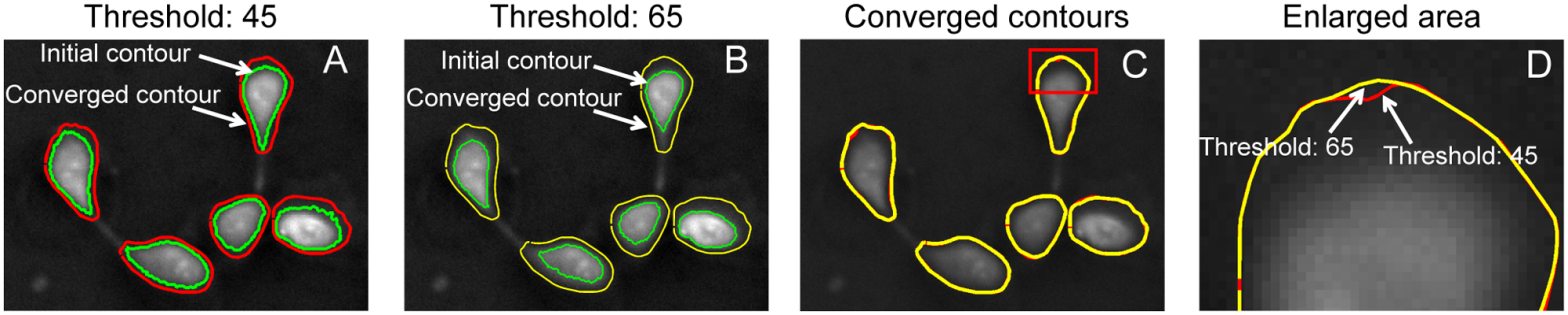
The contour expansion operation is robust to the selected threshold value. The contour expansion was performed with the initial contours obtained with threshold value of 45 (A) and 65 (B). The final converged contours are close to each other, as shown in (C), which implies that the contour expansion operation is robust with respect to the selection of threshold value. (D) The enlarged image for the selected area in (C).

In the thresholding method, the obtained contours are sensitive to the selection of the threshold value. Since the thresholding method was used to determine the initial contours in this study, the influence of the selection of the threshold value on the final converged contour is tested, as shown in Fig 9. Fig 9A and 9B show the cell boundary detection with threshold values of 45 and 65, respectively. One can see that the initial contours obtained with the threshold value of 65 are much smaller than that obtained with the threshold value of 45. However, the converged contours are close to each other, as shown in Fig 9C and 9D. This is because the gradient of light intensity dominates the convergence of the contours and the final positions where the contours stop is mainly determined by the light intensity.

**Fig 10:**
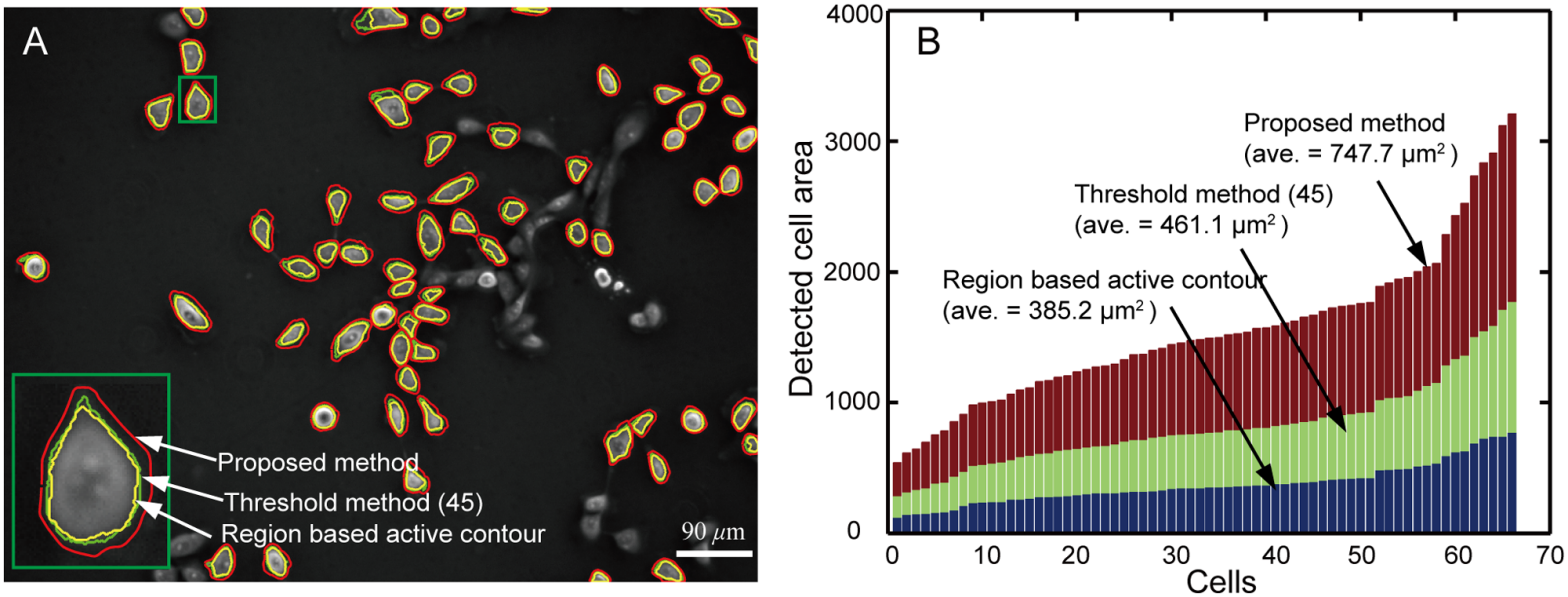
Comparison of boundary detection obtained with different methods for isolated cells. (A) The boundaries detected with the thresholding method, region based active contour method, and the contour expansion method for all isolated cells in the field of view. (B) Comparison of the areas enclosed by the contours detected with different methods. The proposed method detects much larger areas than the other two methods.

A comparison of cell image segmentation results obtained with the thresholding method (threshold value: 45), region based active contour method, and the proposed method is implemented for all isolated cells in the field of view, as shown in Fig 10A. The inset is the enlarged display of the area selected by a green box. One can see that the boundaries detected with the thresholding method and the region based active contour method are close to each other. The boundaries detected by the proposed method enclose larger areas and provide improved estimation of cell boundaries. The comparison of the areas enclosed by the detected boundaries through different methods is shown in Fig 10B. The cells are numbered with increasing areas in the figure. The average area obtained with the proposed method is 747.7 *μ*m^2^, which is much larger than that of 461.1 *μ*m^2^ and 385.2 *μ*m^2^ obtained with the thresholding method and the region based active contour method, respectively.

**Fig 11:**
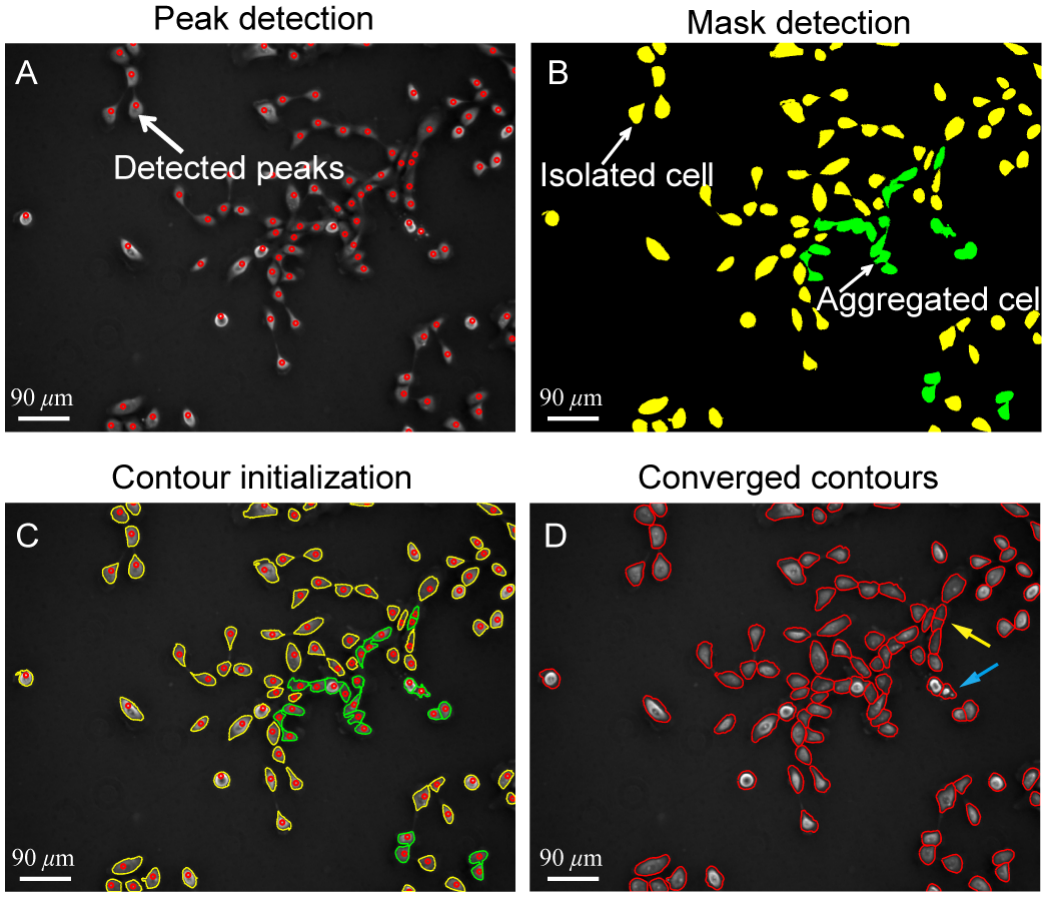
Demonstration of cell localization, boundary detection, and segmentation of the clustered cells. (A) For the negative phase contrast image, peaks of light intensity are detected for all cells, as indicated by red circles. (B) Preliminary masks are obtained with the thresholding method. Masks in green are areas with multiple peaks indicating clustered cells. Masks in yellow are areas with single cell. (C) The boundaries of the preliminarily detected masks are extracted to serve as initial contours for individual cells. (D) Contour expansion method is applied to detect cell boundaries for all cells in the-field-of-view. Except one oversegmentation (marked by a yellow arrow) and one falsely detected cell from a debris (marked by a blue arrow), all the other cells are successfully segmented.

With the proposed methods for cell boundary detection and segmentation of the clustered cells, the cell image segmentation can then be implemented to the negative phase contrast images. Fig 11A shows the raw negative phase contrast image with detected peaks. The thresholding method was then applied to the image and a mask map is obtained, as shown in Fig 11B. In the figure, the yellow masks are the ones with single cells, while the green ones are masks containing multiple cells. The boundaries of the yellow mask areas were directly extracted and taken as the initial contours for contour expansion operation. The green mask areas were first divided into several subareas. After that, the contours for each subarea were extracted (Fig 11C) and the contour expansion method was implemented. The cell boundary detection result is shown in Fig 11D. Almost all cells in the image were successfully segmented, except that an elongated cell was oversegmented as two and a piece of debris was falsely detected as a cell, which is almost inevitable in cell image segmentation, as pointed by yellow and blue arrows in Fig 11D, respectively.

**Fig 12:**
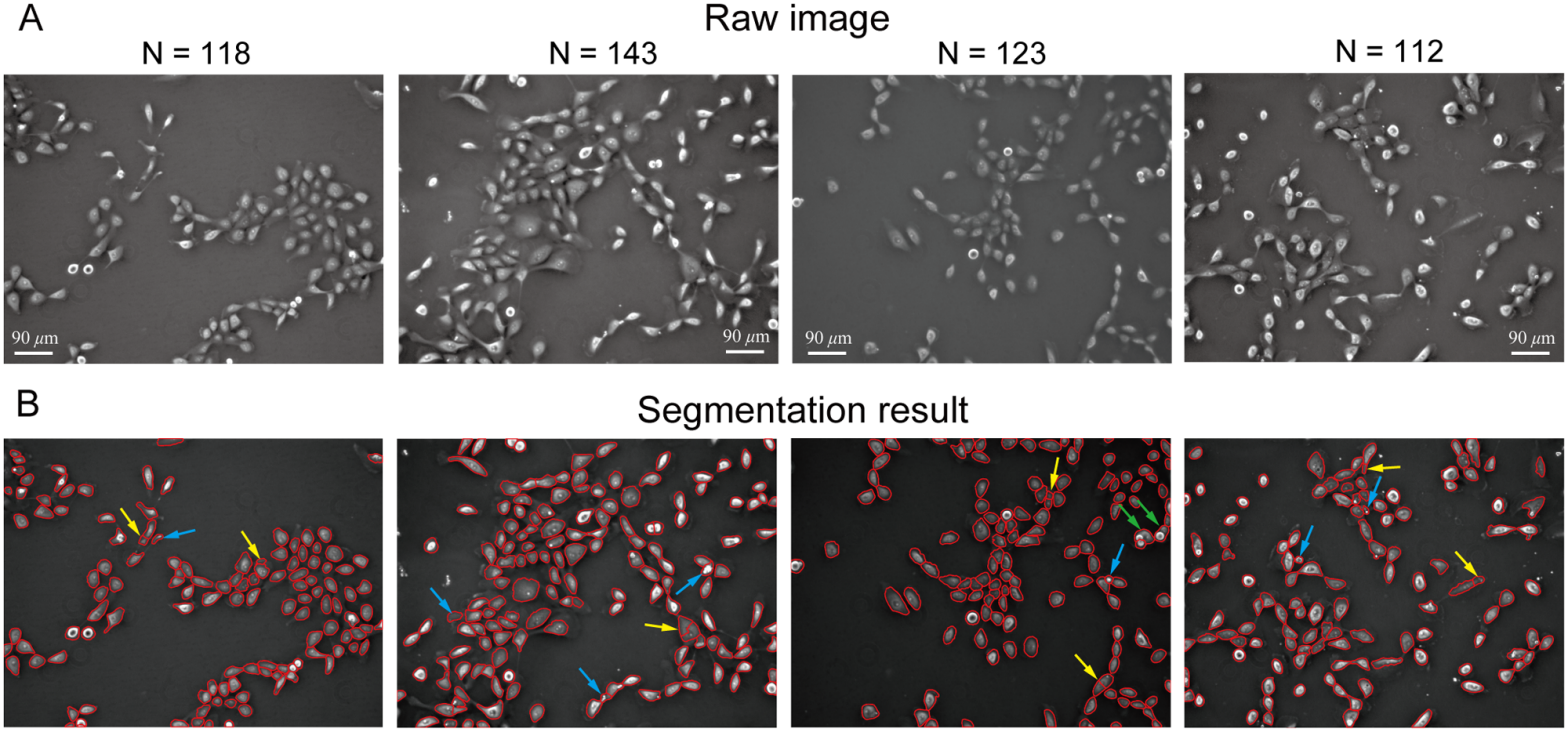
Cell image segmentation result for phase contrast images from four different experiments. (A) Raw negative phase contrast images. (B) Segmentation result with false segmented cells pointed by arrows.

The performance of the proposed method was evaluated with the negative phase contrast images obtained from four different experiments. Fig 12A shows the raw phase contrast images from the experiments. Cell image segmentation was implemented with the proposed method and the results are shown in Fig 12B. The accuracy rate of cell image segmentation is analyzed. In this study, two kinds of false segmentations: oversegmentation and undersegmentation, are considered. In Fig 12B, the oversegmentation and undersegmentation were marked by yellow and green arrows, respectively. Table 1 summarizes the false segmentation rate for all the cells detected in Figs 11 and 12. There are totally 496 cells included in these images. The number for the oversegmentation and undersegmenation is 7 and 2, respectively, corresponding to 1.4% and 0.4% false segmentation rate. The overall false segmentation rate is about 1.8%. Authors want to note here that the performance of the proposed cell segmentation algorithms is supposed to be related to cell density in the field of view. The increased cell density has two impacts on the performance of cell segmentation. One is that it can reduce cell image contrast which leads to false segmentation. The other is that the boundaries between contacting cells will become blurry with increased cell density. This will make it difficult to the extract cell boundaries between any two contacting cells. In addition to oversegmentation and undersegmentation, the debris present in the field of view could be falsely detected as cells, as pointed by blue arrows in Fig 12B. The debris may have the similar size with the cells. Therefore, it is difficult to avoid such kind of false detection.

**Table 1:** Summary of the false segmentation rates obtained with the proposed method.

Total cell number	Over segmentation^£^	Under segmentation^†^	Over detection^‡^	Overall
496	7 (1.4%)	2 (0.4%)	7 (1.4%)	16 (3.2%)

The values in parenthesis are corresponding percentages.

Among the three cell lines, MCF 10A cells have the lowest overall false tracking rate.

^**£**^ Oversegmentation: the number of detected cells is more than their actual number in a given area;

^**†**^ Undersegmentation: the number of detected cells is less than their actual number in a given area;

^**‡**^ Over detection: the debris or artifacts present in the field of view are falsely detected as cells.

## Conclusion

In this study, we have established a program for automated cell image segmentation. The negative phase contrast images were applied to obtain a consistent image contrast for all cells with different cell height in the field of view. The effort was put on the optimization of boundary detection for all cells in the field of view and segmentation of the clustered cells. In cell boundary detection, the thresholding method and a modified edge based active contour method, which is referred to as contour expansion method, were combined to achieve the optimized boundary detection. Driven by the field of gradient of light intensity, the initialized contours determined with the thresholding method converge towards cell boundary in the operation of the contour expansion method. In the segmentation of the clustered cells, the geographic peaks of light intensity were used to determine the numbers and locations of multiple cells. The approach provides accurate estimation of cell locations and boundaries for the clustered cells. The influence of the parameters in contour expansion operation and the selection of the threshold value on the final segmentation results were investigated. The result shows that the proposed method is robust to the selection of threshold values. The relative low values of the two gains in the energy function of parametric contours need to be selected to make sure the appropriate convergence of the contours toward cell boundaries.

The proposed method was validated through automated segmentation of negative phase contrast images from different experiments. The results show that the proposed method can provide optimized cell boundary detection. The average cell area detected by the proposed method is 747.7 μm2, which is much larger than that of 461.1 μm2 and 385.2 μm2 obtained with the thresholding and region based active contour methods, respectively. Moreover, the clustered cells can be well segmented with the proposed method. The method was applied to four cell images containing about 500 cells. Relative low false tracking rates of 1.4% and 0.4% are obtained for oversegmentation and undersegmentation, respectively.

## Supporting Information

S1 Fig Figure of Euclidean Distance Transform for Fig 5B. (TIF)

S1 File Numerical Solution to the active contour model. The file shows details procedure for numerical solution to the edge based active contour model of Eqs (3) and (4).
(DOC)

S1 Zip File Matlab tool box. The developed MATLAB tool box for image segmentation using the method proposed in this paper. The raw images shown in Figs 11 and 12 are also included in the folder.
(ZIP)
