# Supplementary figures and images for: Segmentation of the Clustered Cells with Optimized Boundary Detection in Negative Phase Contrast Images
Source: PLoS One. 2015 Jun 12;10(6):e0130178. doi: 10.1371/journal.pone.0130178 (PMC4467081; doi:10.1371/journal.pone.0130178)

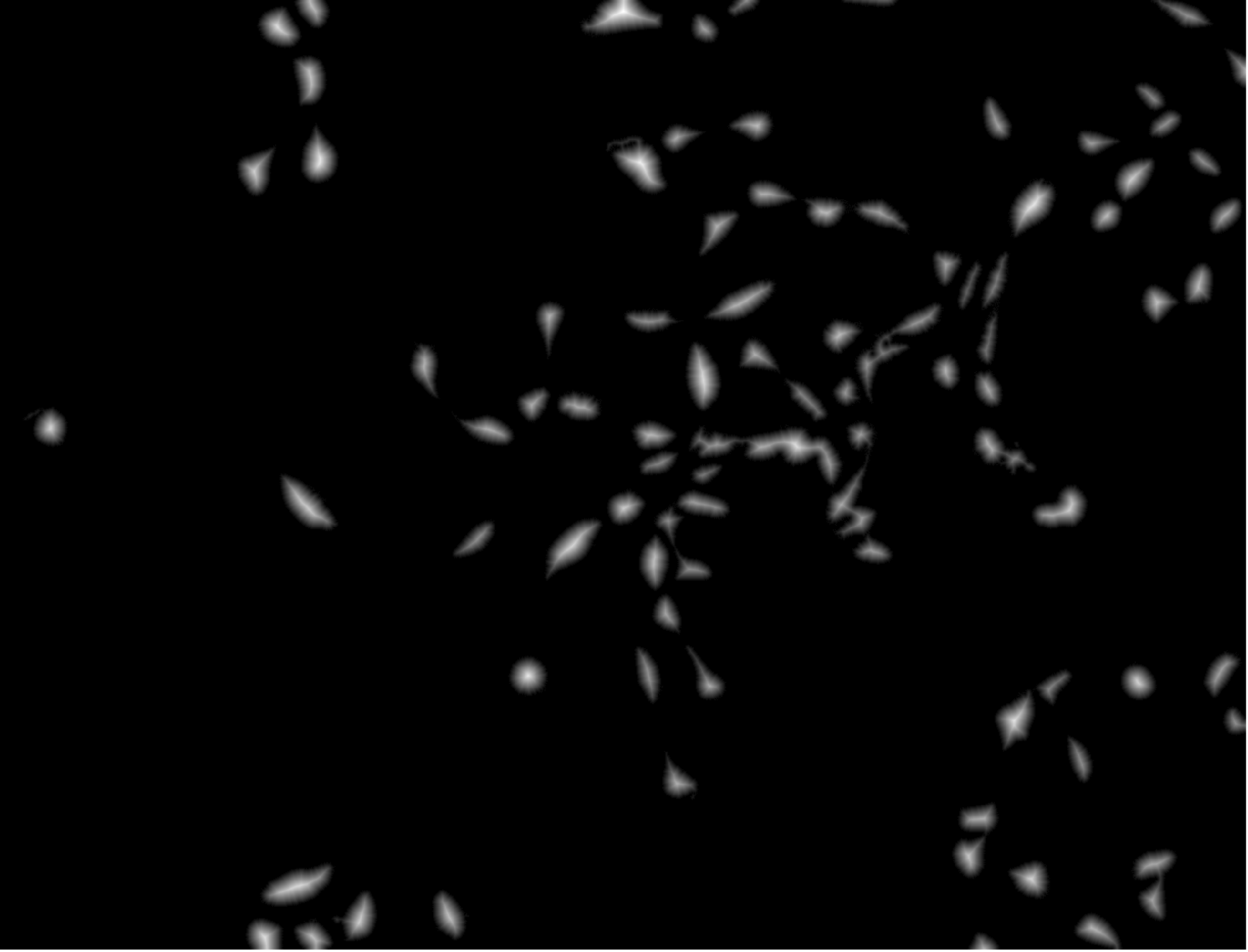

Supplement: S1 Fig — (TIF) [file pone.0130178.s001.tif]

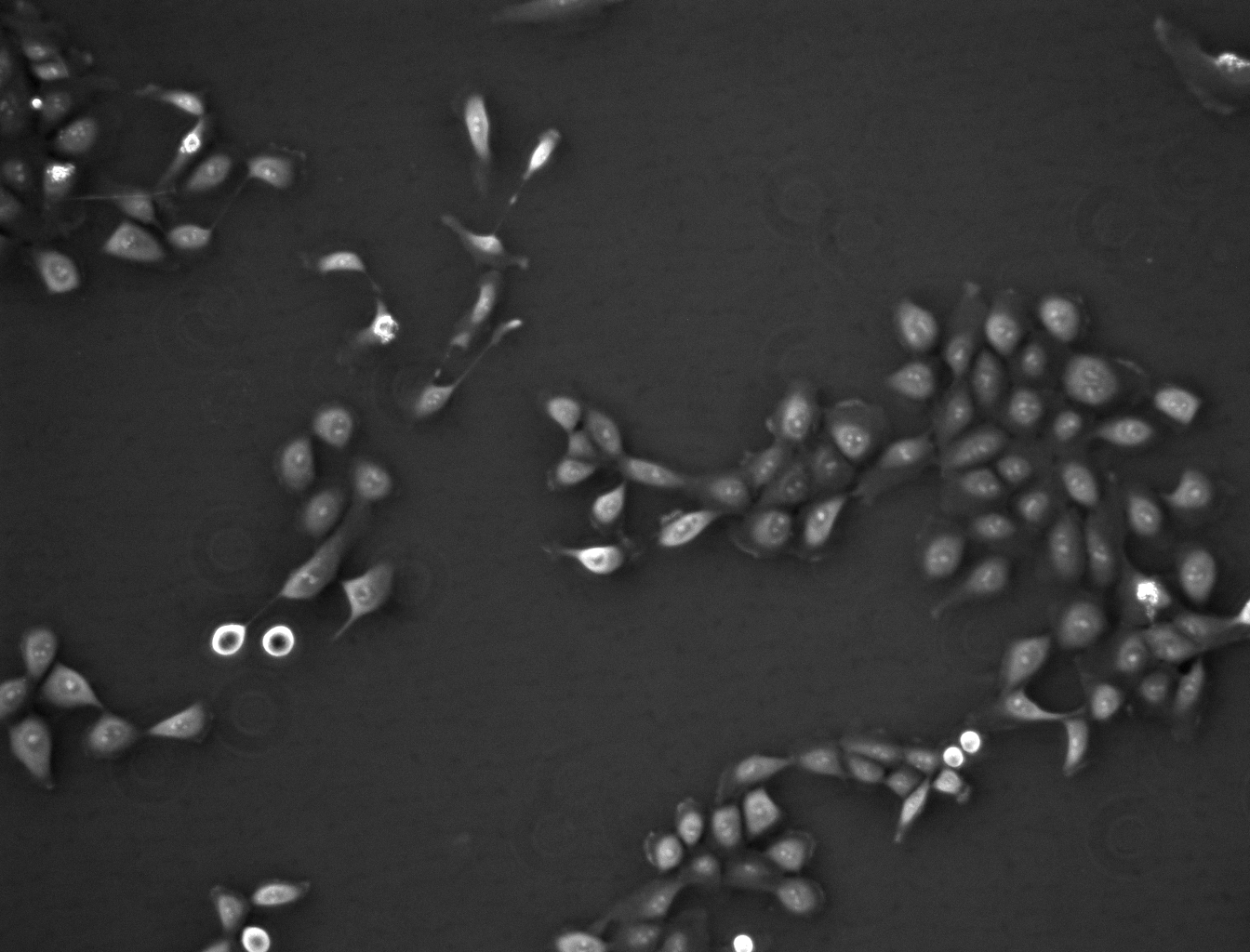

Supplement: S1 Zip File — The developed MATLAB tool box for image segmentation using the method proposed in this paper. The raw images shown in Figs 11 and 12 are also included in the folder. (ZIP) [file pone.0130178.s003.zip › Matlab tool box/Fig 12_1.tif]

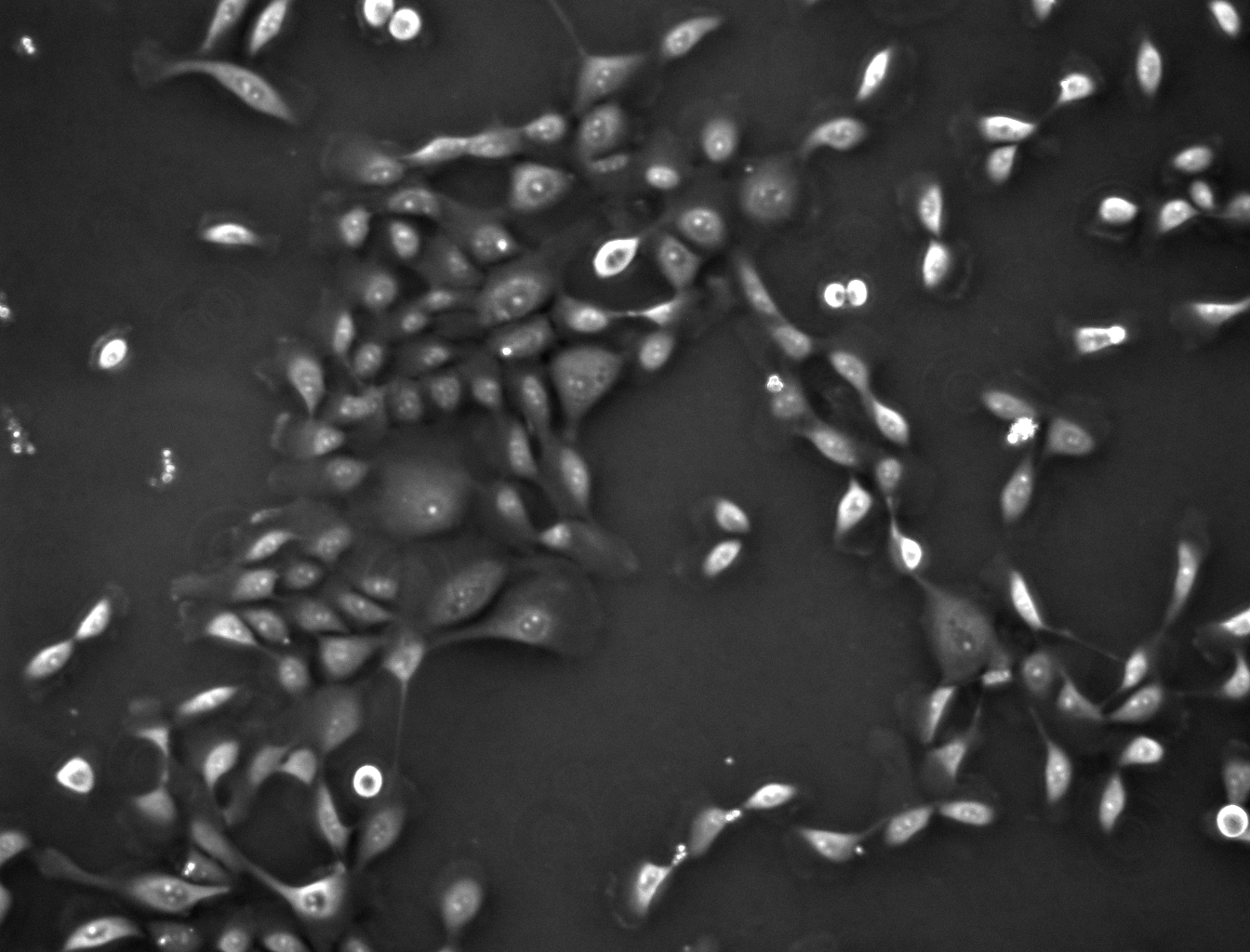

Supplement: S1 Zip File — The developed MATLAB tool box for image segmentation using the method proposed in this paper. The raw images shown in Figs 11 and 12 are also included in the folder. (ZIP) [file pone.0130178.s003.zip › Matlab tool box/Fig 12_2.tif]

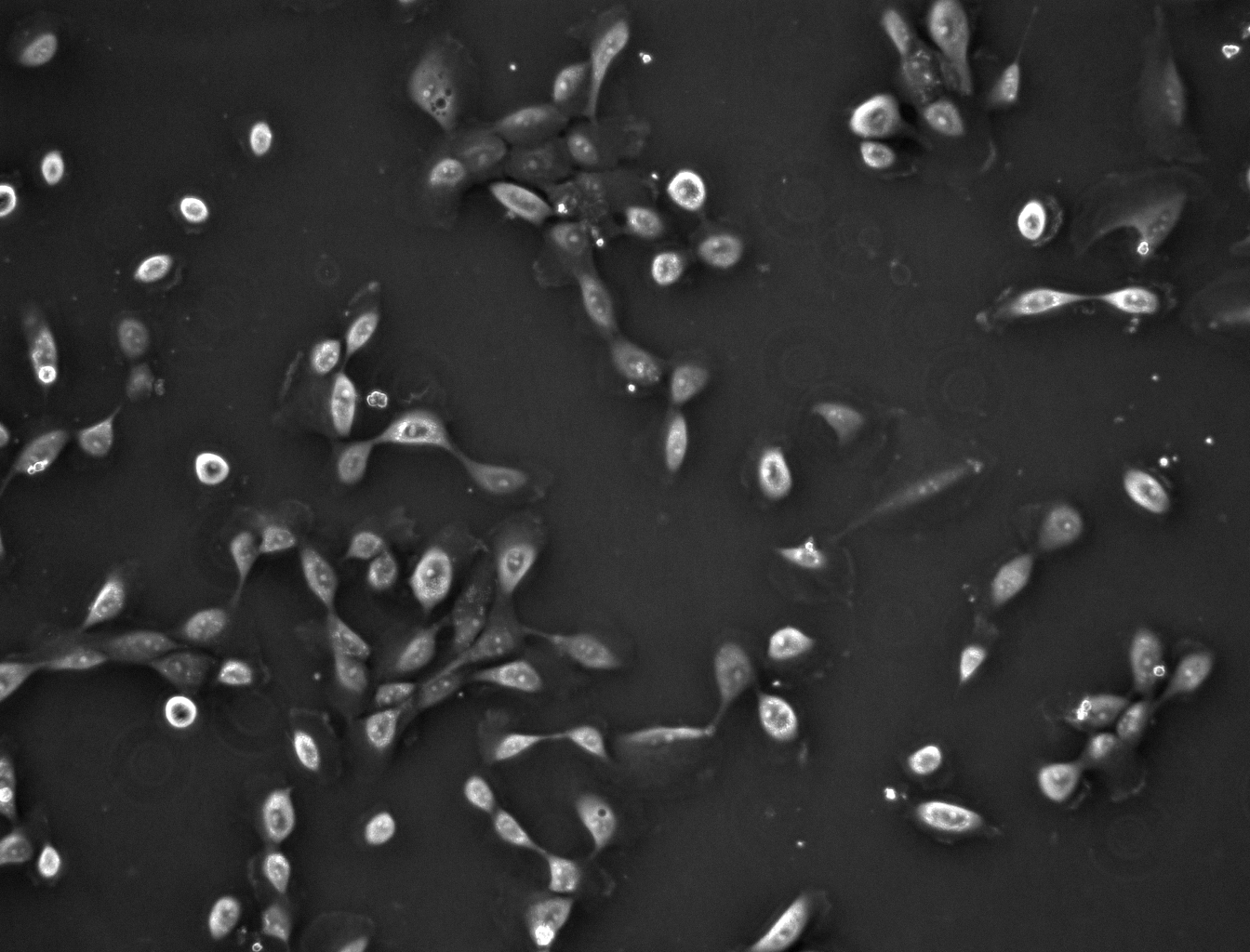

Supplement: S1 Zip File — The developed MATLAB tool box for image segmentation using the method proposed in this paper. The raw images shown in Figs 11 and 12 are also included in the folder. (ZIP) [file pone.0130178.s003.zip › Matlab tool box/Fig 12_4.tif]
