# Supplementary material for: Segmentation of the Clustered Cells with Optimized Boundary Detection in Negative Phase Contrast Images
Source: PLoS One. 2015 Jun 12;10(6):e0130178. doi: 10.1371/journal.pone.0130178 (PMC4467081; doi:10.1371/journal.pone.0130178)
Supplement: S1 File — The file shows details procedure for numerical solution to the edge based active contour model of Eqs (3) and (4). (DOC) [file pone.0130178.s002.doc]

**Numerical Solution to the active contour model**

**for**

**Segmentation of the Clustered Cells with Optimized Boundary Detection in Negative Phase Contrast Images**

Yuliang Wang^1^, [[1]](#footnote-2) , Zaicheng Zhang^1^, Huimin Wang^2^, and Shusheng Bi^1^

*^1^Robotics Institute, School of Mechanical Engineering and Automation, Beihang University, Beijing 100191, P.R. China*

*^2^Department of Materials Science and Engineering, The Ohio State University, 2041 College Rd., Columbus, OH 43210, USA*

**Corresponding author: Yuliang Wang**

**Email: wangyuliang@buaa.edu.cn**

This supplementary material provides a detailed procedure for the numerical solution to the Eq. (4) in the manuscript. One can also see the appendix section in the reference (Kass M et al., International Journal of Computer Vision, Vol. 1, pages:321-331) for details.

In the traditional active contour model, a contour in an image is defined as a parametric contour ***v***(*s*) = (*x*(*s*), *y*(*s*)) and has an energy form given as:

$E=\int_{0}^{1} \left(\frac{1}{2}\alpha v_{s}^{2}+\frac{1}{2}\beta v_{ss}^{2}+E_{ext}\right)ds$, (s1)

where ***v****_s_* and ***v****_ss_* are the first and second order partial derivatives, and *α* and *β* are scalar coefficients. The first two terms in the right side of the **Eq. s1** is the internal energy of the contour, while the *E*_ext_ represents the external energy of the contour. Here, the height of the NBs along the contour is taken as the external energy. The internal energy depends only on the curve geometry and enforces the continuity and certain smoothness of the curve. The minimization of the total energy *E* satisfies the associated Eular-Lagrange function, given as:

$\alpha v_{ss}(s,t)-\beta v_{ssss}(s,t)-\nabla E_{ext}=0$, (s2)

where ***v****_ssss_* is the fourth order partial derivatives of ***v***(*s*).

The **Eq. s2** can be numerically solved. The discrete form of the contour ***v***(*s*) can be expressed as a series of points along the contour, given as *v_i_* = (*x_i_*, *y_i_*) = (*x*(*ih*), *y*(*ih*)), where *h* is the finite step size along the contour. By approximating the derivatives with finite differences, the terms ***v****_ss_* and ***v****_ssss_* at point *i* in **Eq. s2** can then be given as:

$v_{ss}(i)=(v_{i+1}-2v_{i}+v_{i-1})/h$ (s3)

$v_{ssss}(i)=(v_{i+2}-4v_{i+1}+6v_{i}-4v_{i-1}+v_{i-2})/h^{4}$. (s4)

Let *f_x_*(*i*) = ∂*E*_ext_ /∂*x_i_* and *f_y_*(*i*) = ∂*E*_ext_ /∂*y_i_*, we have ▽*E*_ext_ =*f_x,y_*(*i*)= (*f_x_*(*i*), *f_y_*(*i*)).

By combining **Eqs. s2-s4** and substituting ▽*E*_ext_ = (*f_x_*(*i*), *f_y_*(*i*)) into **Eq. s2**, the finite difference form of **Eq. s2** can be given as:

$\beta v_{i+2}-\text{（}\alpha+4\beta\text{）}v_{i+1}+\left(2\alpha+6\beta\right)v_{i}-\left(\alpha+4\beta\right)v_{i-1}+\beta v_{i-2}+(f_{x}(i),f_{y}(i))=0$ (s5)

The above finite difference form Eular-Lagrange function can be written in matrix form as

**A*v*_s_**+ ***f_x,y_***=0 (s6)

where A is a pentadiagonal banded matrix. The **Eq. s6** can be solved through an explicit Euler method between two successive instantaneous time points *t* and *t*-1, given as:

**A*v*_s_**(*t*)+ ***f_x,y_***(*t*-1) = -*γ*(***v_s_***(*t*)-**v*_s_***(*t*-1)) (s7)

where *γ* is a step size. The **Eq. s7** can be solved as:

***v*_s_**(*t*) = (A+ *γ*I)( *γ* ***v*_s_**(*t*-1)-***f_x,y_***(*t*-1)) (s8)

By iteratively solving the **Eq. s8**, the contour will be deformed and converged towards the NB boundary, where the total energy of the contour is minimized.

1. Corresponding author: wangyuliang@buaa.edu.cn [↑](#footnote-ref-2)
